# The role of sex and gender in the selection of Alzheimer patients for clinical trial pre-screening

**DOI:** 10.1186/s13195-021-00833-4

**Published:** 2021-05-05

**Authors:** Maitee Rosende-Roca, Carla Abdelnour, Ester Esteban, Juan Pablo Tartari, Emilio Alarcon, Juliana Martínez-Atienza, Antonio González-Pérez, María E. Sáez, Asunción Lafuente, Mar Buendía, Ana Pancho, Nuria Aguilera, Marta Ibarria, Susana Diego, Sara Jofresa, Isabel Hernández, Rogelio López, Miren Jone Gurruchaga, Lluís Tárraga, Sergi Valero, Agustín Ruiz, Marta Marquié, Mercè Boada

**Affiliations:** 1 Research Center and Memory Clinic. Fundació ACE. Institut Català de Neurociències Aplicades. Universitat Internacional de Catalunya, Gran Vía de Carles III, 85 BIS, 08028 Barcelona, Spain; 2 Networking Research Center on Neurodegenerative Diseases (CIBERNED), Instituto de Salud Carlos III, Madrid, Spain

**Keywords:** Alzheimer’s disease, Dementia, Mild cognitive impairment, Sex, Gender, Women, Aging, Education, Elderly

## Abstract

**Background:**

Alzheimer disease (AD) is a progressive neurodegenerative disorder affecting the elderly with a prevalence of 7.1% in women and 3.3% in men. Sex-related patterns have been reported in prognosis, biomarker status, and risk factors. Despite this, the interaction of sex has received limited attention, with AD trials persistently recruiting lower numbers of women than the population distribution and a lack of information on the sex-disaggregated effects of anti-dementia therapies. This is the first study aiming to identify the role of sex in the selection for screening in AD clinical trials.

**Methods:**

This cross-sectional study provides a comprehensive analysis of screening eligibility according to a set of pre-selection criteria currently applied at Fundació ACE memory clinic for a more efficient trial screening process. A cohort of 6667 women and 2926 men diagnosed with AD dementia (55%) or mild cognitive impairment (45%) was analyzed. We also assessed the frequencies of men and women effectively screened for trial enrolment over a period of 10 years. Additionally, data from AddNeuroMed study was used to explore trends in eligibility based on the education criteria.

**Results:**

Women showed a significantly lower chance of being eligible for screening than men (OR = 1.26; *p* < 0.01). This imbalance was confirmed by a lower frequency of women screened for enrolment compared to the study population (63.0% vs. 69.5%). Education was revealed as the key criterion contributing to this unbalance, with men showing over twice the chance of being screened compared with women (OR = 2.25, *p* < 0.01). Education-based differences were greater in earlier born patients, but the gap narrowed and achieved balance with increasing year of birth. This observation was replicated using data from other European populations included in AddNeuroMed study. Comorbidity was the most limiting criterion with sex differences in frequencies and significant discrimination against the selection of men (OR = 0.86, *p* < 0.01).

**Conclusions:**

The large number of low-educated elderly women with AD demands for a sex-focused approach in clinical research. New assessment tools insensitive to education level should be developed to enable a proportional representation of women. Although this gender education gap is mostly inexistent in developed countries, economic or cultural factors may lead to different scenarios in other regions. Overlooking the impact of sex may lead to a handicap in AD research with a direct adverse impact on women’s health.

**Supplementary Information:**

The online version contains supplementary material available at 10.1186/s13195-021-00833-4.

## Background

Alzheimer disease (AD) is a progressive neurodegenerative disorder causing memory loss, cognitive deficits, and behavioral changes. The hallmark physiopathological features of AD include β-amyloid plaques, neurofibrillary tangles, and neuronal lesions that cause a disruption of metabolic processes leading to a progressive cognition impairment [1]. AD represents 60–80% of all dementia cases, with an estimated overall prevalence of 4.4% among individuals aged 65 years and older [2]. Prevalence increases with advancing age (0.97% for 65–74 years, 7.7% for 75–84 years, and 22.5% for ≥ 85 years) and is significantly higher in elderly women (7.1% in females vs. 3.3% in males) [3]. AD and other dementias are the fifth leading cause of death, killing 2.4 million people globally [4]. In Spain, AD caused a total of 14,929 deaths in 2018, with 10,475 of them occurring in women, accounting for a 5% of overall female mortality [5]. Mild cognitive impairment (MCI) is a transitional clinical entity between normal aging and dementia [6], with about 10–15% of all cases progressing yearly to clinically probable Alzheimer dementia [7]. MCI prevalence ranges from 16 to 20% in patients aged 50 or older, but as opposed to dementia, the evidence of any gender-based differences is unclear [8]. Because the incidence of AD is strongly associated with age, it is expected that population aging as well as the current lack of effective disease-modifying strategies will contribute to an increasing trend in its prevalence that will pose huge challenges to public healthcare systems across the world [9].

Sex- or gender-based differences in the prevalence of AD are not entirely explained by the increased longevity of women, but also by biological or sociocultural differences found between men and women that account for heterogeneity in risk factors, cognitive decline, prognosis, and drug effects [10–12]. The AD phenotype and progression pattern is affected by well-known sex-related (referring to biological variations among men and women) as well as gender-related differences (referring to psychosocial and cultural disparities between males and females) with crucial implications for diagnosis, treatment and clinical research [13].

Sex-specific patterns were reported in the rate of cognitive decline and brain atrophy, in the effects of risk factors as well as in the patterns of diagnostic biomarkers [12]. This variability in disease presentation might indicate differential neuropathological mechanisms operating in men and women. In this regard, women with AD report faster hippocampal atrophy rates and higher prevalence of neurofibrillary tangles and amyloid plaques [14]. The APOE4 allele for instance, a genetic factor for late-onset AD, confers greater risk for developing the disease in women. Hormonal changes (mainly estrogens) linked to woman’s reproductive system, as well as the excess risk of thyroid disease observed in women, have also been associated with higher risk of AD [15]. Sex differences were also found in the rates comorbidities and the use of drugs in AD patients. Depression, anxiety, thyroid disease, autoimmune disorders, and chronic pain, which in turn lead to the use of psychotropics, hormonal drugs, immunosuppressants, or opioids among others, were more frequently reported in women [12]. These conditions often involve a disruptive effect on cognitive function and consequently lead to a higher risk or a worse prognosis of AD. Furthermore, understanding the interaction of sex on the effects of anti-dementia drugs may also have important implications for women’s health. Anti-dementia therapies with suboptimal safety/efficacy evidence in women may in fact increase the risk of poorer outcomes or adverse effects in the female population with AD. However, despite this, few studies thus far provide sex-disaggregated data [16].

A well-known gender-related factor affecting AD is cognitive activity. Low cognitive activity has been associated with a higher risk of developing of AD [17] as well as a longer duration of the disease [18]. In the past, men have had more opportunities for better education and higher occupational status than women, and thus, particularly in the older aged groups (≥ 70 years), women are at higher risk of presenting AD [19]. However, intellectual lifestyles in women have changed which may transform the epidemiological patterns of dementia in the near future. Another gender-associated factor indirectly reverting the burden of AD on women is the use of caregivers. An estimated 71% of all dementia patients have a caregiver [20], and approximately two thirds of them are women [1].

Women underrepresentation in clinical research is a persistent problem according to a study involving 43,000 published research studies and 13,000 registered clinical trials over 25 years [21]. Average women enrolment across studies is close to 50%; however, for many disease types, women participation is not proportional to the burden of the disease. Furthermore, according to a report from the Spanish Agency of Medicines, only 20% of the trials present sex-disaggregated efficacy or safety data [22]. In a systematic review considering 48 AD studies and a total of 20,688 patients, the overall proportion of women was 63.8%, with nearly all trials recruiting a larger number of women, but minimal information on the potential effect of sex on treatment efficacy or tolerability [16]. According to this report, women participation mirrored the sexually unbalanced prevalence of AD; however, this estimate was below that found the population living with the disease. Previous reports also revealed significant discrepancies in the gender distributions of AD trial participants and the general population (63.2% versus 67.8% respectively) [23]. The representativeness of women in AD research is therefore arguable, more importantly because they are the primary users of anti-dementia drugs. A proportional participation of women in clinical trials is key to warrant the external validity of findings, allowing for a better understanding of drug effects in men and women, ultimately leading to improved tolerability and clinical outcomes. However, thus far, the relevance of sex as interacting factor on the efficacy and safety of anti-dementia therapies has received limited attention with scarce sex-disaggregated evidence on the effects of drugs [16] and no reports to date analyzing the potential causes of gender imbalances in trial enrolment.

Building on this background, we have designed a cross-sectional study aiming to identify the role of sex in the eligibility for participation in dementia trials and to analyze the causes of any found disparities between men and women. To this end, a cohort of 9593 patients with AD dementia or MCI was analyzed according to a set of pre-screening criteria currently applied at Fundació ACE memory clinic for a more efficient trial enrolment process. The distribution of men and women screened for trial enrolment over a period of 10 years was also assessed.

## Methods

### Study design, participants, and settings

**Fig. 1:**
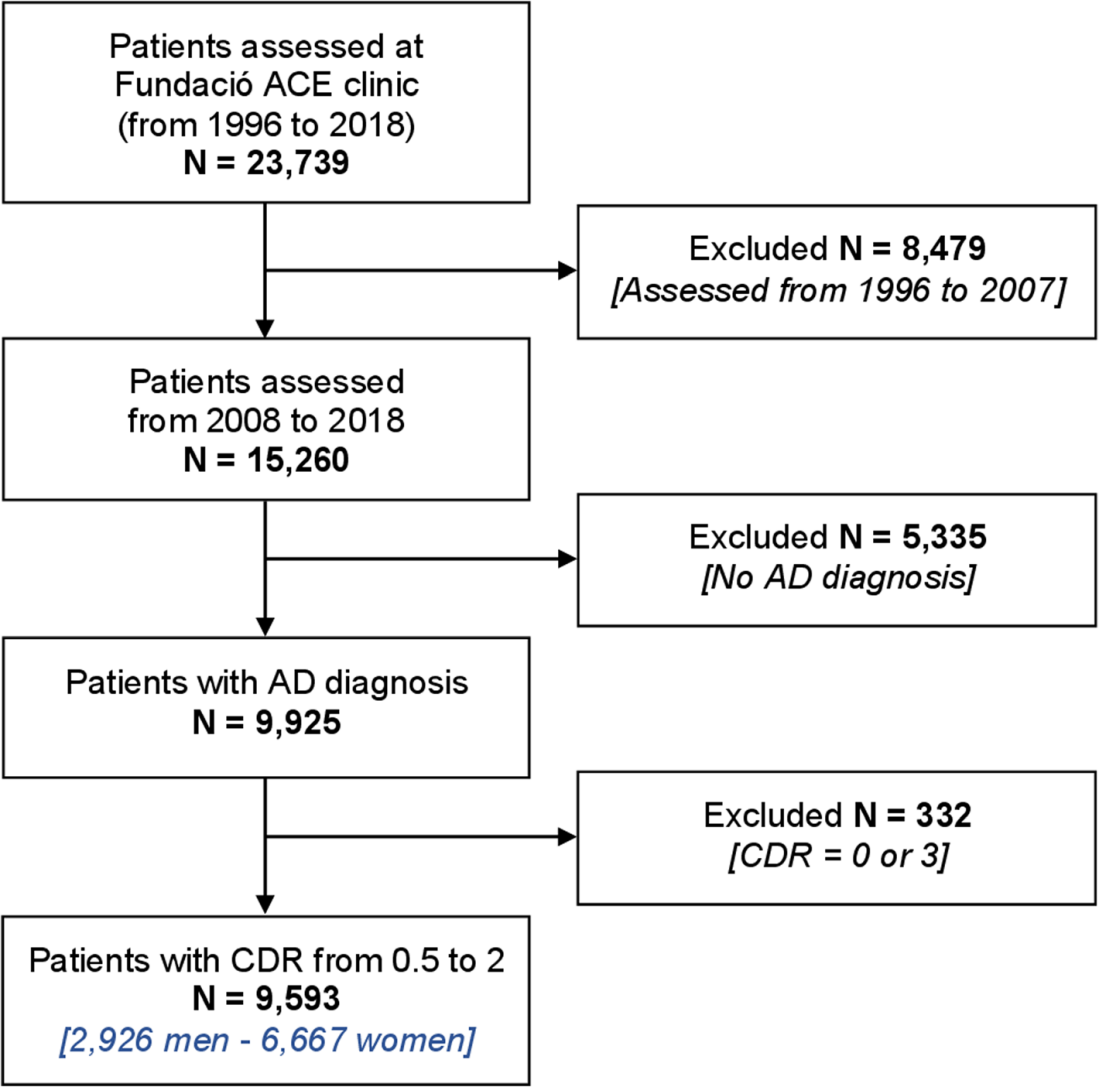
Cohort selection flow diagram. The study cohort was selected among a total population of 23,739 subjects assessed at Fundació ACE clinic according to three selection requisites: (1) assessment performed from 2008 to 2018, (2) confirmed initial diagnosis of AD (either dementia or mild cognitive impairment) and clinical dementia rating (CRD) score of 0.5-2. The resulting study sample comprised a total of 9593 subjects, 2926 men and 6667 women

This a cross-sectional study evaluating a cohort of 9593 patients with an initial diagnosis of AD dementia (5278 subjects) or MCI (4315 subjects) with a Clinical Dementia Rating (CDR) score of 0.5 to 2, admitted to Fundació ACE - Alzheimer Research Center and Memory Clinic (in Barcelona, Spain) from 2008 to 2018. Subjects with cognitive impairment resulting from causes other than AD were excluded (vascular dementia, frontotemporal degeneration or dementia with Lewy bodies). The study cohort was selected according to these criteria among a total population of 23,739 subjects assessed at Fundació ACE memory clinic (Fig. 1). Exclusions from the study cohort in terms of the year of assessment (a total of 8479 patients) were not expected to cause any bias in the results. Data collected refers to the information registered for each subject during admission to the clinic. Variables analyzed were sex, year of birth, education level, cognitive function according to the mini-mental state score (MMSE), and predementia syndrome diagnosis. We also assessed the prevalence of comorbidities and the use of concomitant drugs that specifically limit trial enrolment (Table 1).

**Table 1:** Exclusion criteria for clinical trial pre-screening eligibility at Fundació ACE

**Age:** age < 50 or > 85 years	
**Comorbidities:** cardiac disorders (such as acute myocardial infarction, chronic heart failure, heart rhythm disorders, syncope or cardiac arrest), Parkinson’s disease, previous history of cerebrovascular disease (stroke, cadasil, cerebral amyloid angiopathy), kidney disease, epilepsy, chronic respiratory disease, severe depression, psychotic disorders, previous history of head trauma with consciousness loss, history of alcohol abuse	
**Concomitant medications:** anti-neoplastic agents including hormone therapy, anti-parkinsonians, anti-psychotics, anti-manic and antiepileptic drugs, anti-rheumatics, anti-coagulants, corticosteroids immunosuppressants, opioids, drugs for alcoholism, psychostimulants	
**Education:** less than 5 years of full-time studies.	
**Mini-mental state examination (MMSE):** score ≤ 12	

These eligibility criteria were defined according to selection requirement commonly observed in dementia trials

Fundació ACE is a unique care clinic that integrates diagnosis, therapy, follow-up care, day care, and day hospital, including a clinical research program that ensure efficient subject screening and meaningful participation in clinical trials [24]. The diagnosis of Alzheimer dementia was based on either the recommendations of the National Institute of Neurological and Communicative Disorders and Stroke and the Alzheimer’s Disease and Related Disorders Association or the National Institute on Aging-Alzheimer's Association criteria [25, 26]. MCI was classified in terms of amnestic and non-amnestic MCI using the current criteria [27–29] and required the presence of measurable alterations in memory and/or cognition, not sufficient to warrant diagnosis of dementia. All diagnoses were assigned by clinical consensus and involved neurobehavioral examinations, including neuropsychological [30] and social work evaluations, as well as appropriate laboratory and neuroimaging studies. Fundació ACE memory unit serves citizens in the catchment area around the city center of Barcelona, providing care through the Catalan Public Health Service, as well as on a fee-for-service basis. From a research perspective, patients treated at Fundació ACE provide a pragmatic sample of the more clinically diverse population with dementia syndromes that are typically seen in clinical care.

### Eligibility for trial screening

The Clinical Trials Unit at Fundació ACE comprises a multidisciplinary team of professionals working to facilitate trial participation in phase II and III clinical trials, either industry funded, or academia initiated, in AD dementia and MCI. In general terms, successful recruitment in clinical trials depends on efficient eligibility screening. This process typically requires manual evaluation of clinical data, which takes a considerable amount of time and effort. Eligibility screening is further complicated by the need to re-assess patients according to disease progression by the detailed selection criteria of each study. To reduce the burden of this process while increasing its efficiency and accuracy, Fundació ACE memory clinic has implemented a pre-screening process that facilitates the identification of participants most likely to meet clinical trial requirements. This devised process is based on set of pre-screening requirements according age, comorbid conditions, concomitant treatments, and MMSE as well as education level (Table 1). These eligibility requirements were designed on the basis of inclusion and exclusion criteria frequently observed in AD trials. A pre-screening list of potentially eligible candidates for screening is generated by the application of this pre-defined set of selection criteria to the clinic’s patient registry, followed by subsequent detailed review of clinical data to confirm whether trial selection criteria were met. Overall, this pioneering trial pre-screening strategy reduces the time and costs of trial selection, by increasing the number of eligible candidates while decreasing the amount of screening failures. Moreover, it can serve as a valuable tool for the analysis of the population of candidates for screening in AD trials.

### AddNeuromed data

We specifically explored trends in eligibility based on the education criteria among men and women by year of birth using data from AddNeuromed study obtained from Synapse. Briefly, this study included a total of 716 individuals (259 AD cases, 225 MCI, and 232 controls) from six different European countries (UK, France, Italy, Greece, Poland, and Finland) [31].

### Analysis and presentation of data

Absolute and relative frequencies of eligible candidates were calculated for each criterion by sex, year of birth, and clinical diagnosis. Multivariate as well as univariate (for all criteria) logistic regression models were used compare eligibility between male and females in each group. The distribution of men and women among eligible candidates, subjects effectively screened for enrolment, and patients in the study population was also estimated. All analyses were performed using Stata 12.1. Graphpad PRISM software 8 was used for the graphical representation of data.

## Results

### Study population

A total of 9593 patients initially diagnosed of AD dementia or MCI (CDR = 0.5–2) at Fundació ACE memory clinic from 2008 to 2018 were selected among a total population of 15,260 medically assessed subjects. The cohort comprised of 2926 men (30.5%) and 6667 women (69.5%) (Fig. 1). AD dementia diagnosis was confirmed for 1499 men (28.4%) and 3812 women (72.2%), whereas a total of 1460 men (33.8%) and 2855 (66.2%) women presented MCI (Table 2). Most patients were born either from 1925 to 1934 (3912; 40.8%) or from 1935 to 1944 (3230; 33.7%) (Table 3). The younger aged group (i.e., those born in 1960 or later) comprised a total of 211 subjects (2.2%; 60 men and 151 women), whereas the older aged group (i.e., those born before 1925) comprised 748 subjects (7.8%; 160 men and 588 women).

**Table 2:** Frequencies of eligible candidates for clinical trial screening by diagnosis and sex

Diagnosis—gender (***N***)	Age	Comorbidity	Medication	MMSE	Education	All criteria
**All (9593)**	7874 (82%)*	3791 (40%)*	**6150 (64%)**	**9269 (97%)**	5789 (60%) *	1458 (15%)*
Men (2926)	2480 (85%)	1106 (38**%**)	1925 (66**%**)	2846 (97**%**)	2144 (73**%**)	506 (17**%**)
Women (6667)	5394 (81%)	2685 (40**%**)	4225 (63**%**)	6423 (96**%**)	3645 (55**%**)	952 (14**%**)
OR [95%CI]	1.22 [1.08–1.37]	0.86 [0.78–0.94]	1.08 [0.98–1.19]	1.09 [0.84–1.42]	2.25 [2.05–2.48]	1.26 [1.12–1.41]
**AD (5278)**	3875 (73%)*	2106 (40%)*	**3222 (61%)**	**4973 (94%)**	2792 (53%) *	623 (12%)*
Men (1466)	1122 (77%)	563 (38**%**)	899 (61**%**)	1393 (95**%**)	984 (67**%**)	208 (14**%**)
Women (3812)	2753 (72%)	1543 (40**%**)	2.323 (61**%**)	3580 (94**%**)	1808 (47**%**)	415 (11**%**)
OR [95%CI]	1.22 [1.06–1.41]	0.87 [0.77–0.99]	0.98 [0.86–1.11]	1.07 [0.81–1.41]	2.26 [1.99–2.57]	1.35 [1.13–1.62]
**MCI (4,315)**	**3999 (93%)**	1685 (39%)*	2928 (68%)*	**4296 (100%)**	2997 (69%) *	**835 (19%)**
Men (1,460)	1358 (93**%**)	543 (37**%**)	1026 (70**%**)	1453 (100**%**)	1160 (79**%**)	298 (20**%**)
Women (2,855)	2641 (93**%**)	1142 (40**%**)	1902 (67**%**)	2843 (100**%**)	1837 (64**%**)	537 (19**%**)
OR [95%CI]	1.05 [0.82–1.34]	0.85 [0.74–0.97]	1.19 [1.04–1.37]	0.81 [0.31–2.11]	2.15 [1.85–2.49]	1.11 [0.95–1.30]

Unless otherwise noted, data are absolute frequency (relative frequency %)

MMSE, mini-mental state examination; AD, Alzheimer’s disease; MCI, mild cognitive impairment

Asterisk (*) indicate *p* < 0.05 in test comparing eligibility between males and females by multivariable logistic regression (or univariable logistic regression for all criteria)

**Table 3:** Frequencies of eligible candidates for clinical trial screening by year of birth and gender

All/YOB-gender (***N***)	Age	Comorbidity	Medication	MMSE	Education	All criteria
**< 1925 (748)**	**97 (13%)**	**295 (39%)**	**435 (58%)**	**694 (93%)**	321 (43%)*	**10 (1%)**
Men (160)	22 (14**%**)	57 (36**%**)	86 (54**%**)	149 (93**%**)	93 (58**%**)	2 (1**%**)
Women (588)	75 (13**%**)	238 (40**%**)	349 (59**%**)	545 (93**%**)	228 (39**%**)	8 (1**%**)
OR [95%CI]	1.18 [0.70–1.99]	0.84 [0.58**–**1.22]	0.80 [0.56**–**1.15]	1.02 [0.50**–**2.05]	2.21 [1.54**–**3.16]	0.92 [0.19**–**4.36]
**1925–1934 (3912)**	**2912 (74%)**	1489 (38%) *	**2486 (64%)**	**3772 (96%)**	1985 (51%)*	**426 (11%)**
Men (1163)	873 (75**%**)	408 (35**%**)	750 (64**%**)	1131 (97**%**)	771 (66**%**)	144 (12**%**)
Women (2749)	2,039 (74**%**)	1081 (39**%**)	1736 (63**%**)	2641 (96**%**)	1,214 (44**%**)	282 (10**%**)
OR [95%CI]	1.11 [0.94**–**1.30]	0.79 [0.68**–**0.92]	1.05 [0.90**–**1.22]	1.14 [0.76**–**1.72]	2.51 [2.17**–**2.90]	1.24 [1.00**–**1.53]
**1935–1944 (3230)**	**3230 (100%)**	1278 (40%) *	**2115 (65%)**	**3148 (97%)**	2,047 (63%)*	611 (19%)*
Men (1019)	1016 (100**%**)	383 (38**%**)	690 (68**%**)	998 (98**%**)	760 (75**%**)	215 (21**%**)
Women (2211)	2211 (100**%**)	895 (40**%**)	1425 (64**%**)	2150 (97**%**)	1287 (58**%**)	396 (18**%**)
OR [95%CI]	1.00 [na]	0.84 [0.72**–**0.98]	1.13 [0.96**–**1.33]	1.14 [0.69**–**1.90]	2.11 [1.79**–**2.49]	1.23 [1.02**–**1.48]
**1945–1959 (1492)**	**1091 (100%)**	**657 (44%)**	**1003 (67%)**	**1451 (97%)**	1238 (83%)*	**383 (26%)**
Men (524)	524 (100**%**)	234 (45**%**)	364 (69**%**)	511 (98**%**)	465 (89**%**)	136 (26**%**)
Women (968)	967 (100**%**)	423 (44**%**)	639 (66**%**)	940 (97**%**)	773 (80**%**)	247 (26**%**)
OR [95%CI]	1.00 [ na]	1.02 [0.82**–**1.26]	1.14 [0.90**–**1.43]	1.12 [0.57**–**2.20]	1.97 [1.44**–**2.69]	1.02 [0.80**–**1.30]
**1960+ (211)**	**144 (68%)**	**72 (34%)**	**111 (53%)**	**204 (97%)**	**198 (94%)**	**21 (13%)**
Men (60)	42 (70**%**)	24 (40**%**)	35 (58**%**)	57 (95**%**)	55 (92**%**)	9 (15**%**)
Women (151)	102 (68**%**)	48 (32**%**)	76 (50**%**)	147 (97**%**)	143 (95**%**)	19 (13**%**)
OR [95%CI]	1.13 [0.58–2.20]	1.36 [0.72**–**2.54]	1.32 [0.72**–**2.44]	0.73 [0.10**–**5.39]	0.72 [0.15**–**3.37]	1.23 [0.52**–**2.89]

YOB, year of birth; MMSE, mini-mental state examination

Unless otherwise noted, data are absolute frequency (relative frequency %)

Asterisk (*) indicate *p* < 0.05 in test comparing eligibility between males and females by multivariable logistic regression (or univariable logistic regression for all criteria)

Among drug classes included in the medication criterion, the most frequently used drug class in women was antipsychotics (10.7%), whereas in men, anticoagulants were the most common (10.5%). The use of anticoagulant drugs was in fact significantly higher in men compared with women (OR = 1.45, *p* < 0.01). On the contrary, women were more likely to be using steroids or opioids drug classes (OR = 0.53 and 0.38 respectively, *p* < 0.01; Supplementary Figure 2). No significant differences were observed in the use of antipsychotic drugs (*p* = 0.17).

The most frequent of the comorbidities included in the criterion were heart disease and depression. Among men, heart disease (31.8%) was the most frequent eligibility-limiting comorbidity, followed by depression (18.1%), alcohol abuse (16.9%), and chronic pulmonary disease (16.5%). In women, the most common disorders causing ineligibility were depression (37.3%) and heart disease (20.8%). Depression was significantly more prevalent in women (*p* < 0.01), while men were more likely to present cardiac disorders, alcohol abuse, pulmonary disease, or kidney disorders (*p* < 0.01) (Supplementary Figure 3). Irrespective of eligibility, the most commonly observed comorbidities in our cohort were osteoarthritis, hypertension and dyslipidemia.

### Eligibility for screening

**Fig. 2:**
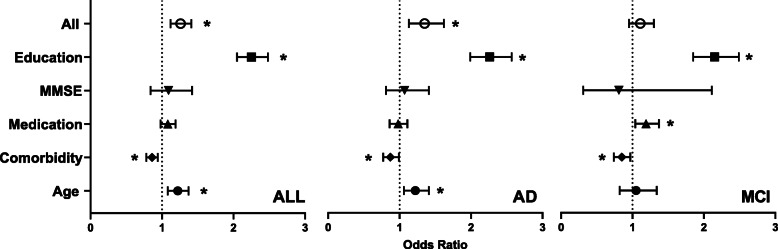
Odds ratio values comparing eligibility in men and women according to pre-screening selection criteria (age, comorbidity, medication, MMSE, education and all criteria) in the study population (ALL), in the patients diagnosed with Alzheimer dementia (AD) and with minor cognitive impairment (MCI). Asterisk (*) indicate *p* < 0.05 in test comparing eligibility between males and females by multivariable logistic regression (or univariable logistic regression for all criteria). MMSE, mini-mental state examination

**Fig. 3:**
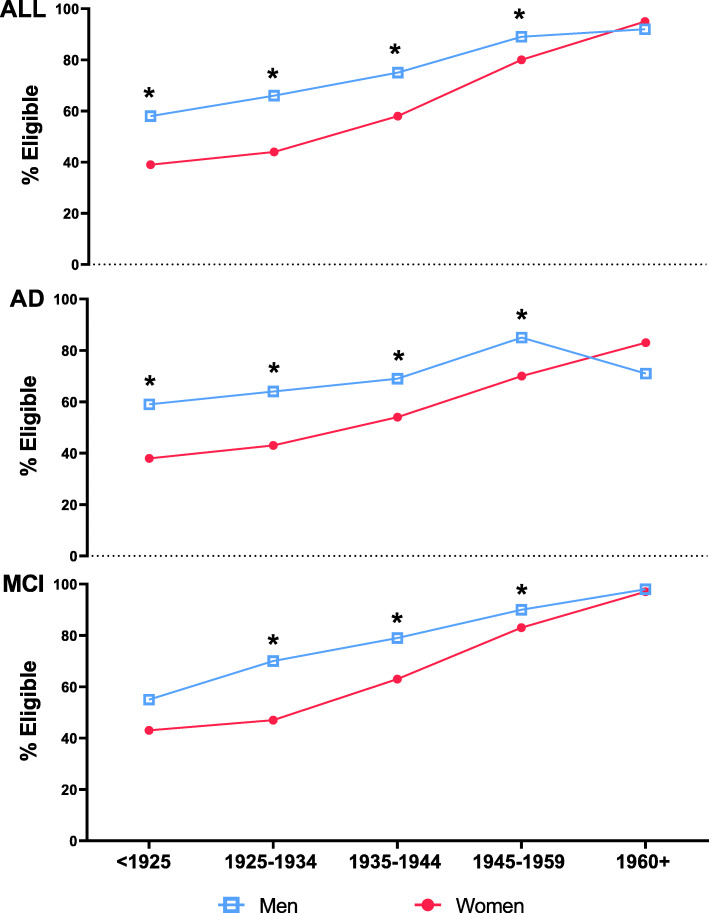
Frequencies of eligible candidates for trial screening according to education by year of birth, sex, and diagnosis, in the study population (ALL), in the patients diagnosed with Alzheimer dementia (AD) and with minor cognitive impairment (MCI). Asterisk (*) indicates *p* < 0.05 in multivariable logistic regression test comparing eligibility between males and female

Exclusion based on education level occurred in 40% of the study population, with marked differences between sexes (Table 2). According to the education requisite, men showed over twice the chance of being screened compared with women (OR = 2.25, *p* < 0.01). This requisite was more restrictive for patients diagnosed with AD dementia compared with MCI (53% vs. 69% eligible candidates respectively); however, the gender-based differences were conserved in both subgroups (OR = 2.26, *p* < 0.01 and OR = 2.15, *p* < 0.01 respectively; Fig. 2). Disaggregated data according to year of birth revealed that the restrictive effect of education and gender imbalances in eligibility were greatest among patients with born before 1925 (43% eligible candidates, OR = 2.21, *p* < 0.01). In fact, the limiting effect as well as the gender gap caused by education declined with increasing year of birth until achieving balance among patients born in 1960 or later (94% eligible candidates, OR = 0.72, *p* = 0.68) (Table 3; Fig. 3). Similar findings were observed in the subgroup analysis according to diagnosis, with the exception of patients with MCI born before 1925, for whom gender differences were non-significant (Fig. 3, supplementary Tables 1 and 2). However, this disparity may be explained by the lower statistical power of this group that would hinder our ability to detect significant differences. Data from AddNeuroMed study confirmed similar large gender differences in eligibility based on the education criteria among those born between 1925 and 1934 (77% vs. 57% in men and women respectively) that tend to decrease among those born between 1935 and 1944 (80% vs. 72%) and become inexistent among those born between 1945 and 1959 (100% vs. 100%)

The presence of comorbidities was the most restrictive criterion, leading to the ineligibility of 60% of the study population (Table 2). According to this requirement, men showed a modest but significantly lower chance of being eligible for screening than women (OR = 0.86, *p* < 0.01). With a similar effect size, this sex-based difference in eligibility was maintained in the groups with Alzheimer dementia and MCI (OR = 0.87, *p* = 0.03 and OR = 0.85, *p* = 0.01 respectively; Fig. 2). The majority of all assessed eligibility-limiting comorbidities occur predominantly in men, which may explain the observed penalizing effect of comorbidities on males. When grouped by year of birth (Table 3), comorbidities-based eligibility was most restrictive for patients born in 1960 or later, with only 34% of eligible candidates. Sex-based differences in eligibility according to the presence of comorbidities were only significant in the largest groups, born from 1925 to1934 and from 1935 to 1944 (OR = 0.79, *p* < 0.01 and OR = 0.84, *p* = 0.03 respectively).

The use of concomitant medication led to the ineligibility of 36% of the study subjects, 39% of patients with AD dementia, and 32% of subjects diagnosed with MCI (Table 2). Sex differences in eligibility according to medication were only significant in patients with MCI, whereby women were penalized in the selection for trial screening (OR = 1.19, *p* = 0.01). When disaggregated by year of birth, this difference in medication-based eligibility was only conserved in patients with MCI born before 1925 (supplementary data Table 2).

Mini-mental state examination score (MMSE) and age were the least restrictive requirements for screening eligibility, allowing for the selection of 97% and 82% of the study population respectively (Table 2). Screening eligibility according to age was significantly higher in women compared with men (OR = 1.22, *p* < 0.01). This age-related sex gap was confirmed for patients with Alzheimer dementia (OR = 1.22, *p* = 0.01), but not in those diagnosed with mild cognitive impairment (Fig. 2). No sex-related differences were found in eligibility according to MMSE (Fig. 2).

After applying all criteria, we found that only 15% of the study population was eligible, with a clear imbalance in eligibility according to sex: women showed significantly lower chance of being eligible for screening than men (OR = 1.26, *p* < 0.01) (Table 2). This sex-based imbalance was confirmed in patients with dementia (OR = 1.35, *p* < 0.01), but not in those in the prodromal stage of AD (*p* = 0.21) (Fig. 2). The limiting effect of this set of criteria was greater in patients with dementia compared to MCI (12% vs. 19% eligible candidates respectively, Table 2). In the analysis by year of birth (Table 3), the effect of the application of all criteria was most limiting in patients born before 1925 (1% eligible candidates); however, the frequency of eligible candidates increased with year of birth up to 26% in the subgroup born from 1945 to 1959. Gender-based differences in screening eligibility were only significant in patients born from 1935 to 1944 (OR = 1.23, *p* = 0.03) and close to significance in those born from 1925 to 1934 (OR = 1.24, *p* = 0.05). Overall, the level of education was the main criterion contributing to this sex imbalance in screening eligibility.

### Gender bias in eligibility for screening

**Fig. 4:**
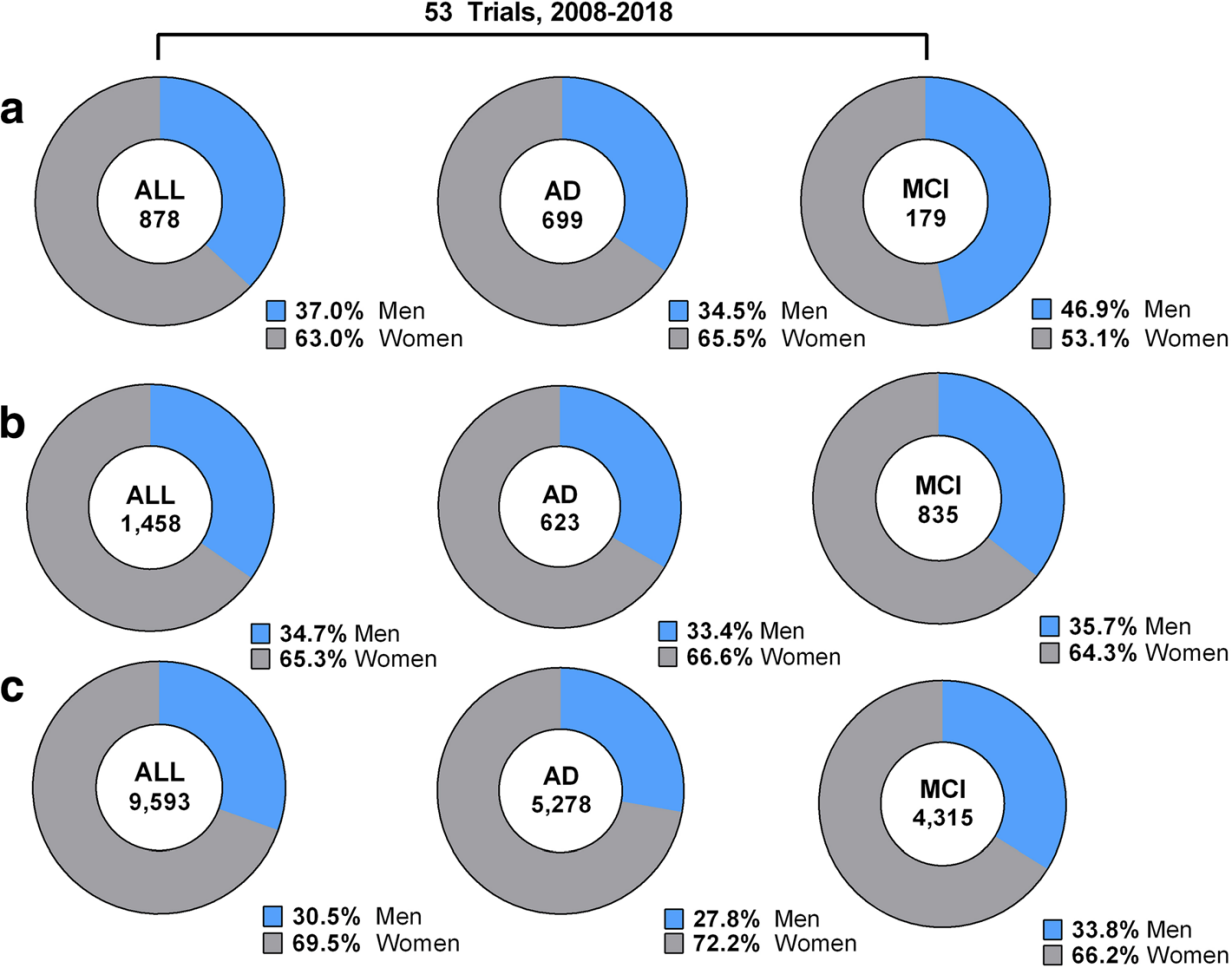
Sex distribution in the sample of patients screened at Fundació ACE memory clinic from 2008 to 2018 (**a**) compared with the distribution in eligible candidates (**b**) and in the study population (**c**) for all the patients (ALL), patients with Alzheimer dementia (AD) and patients with minor cognitive impairment (MCI)

In general terms, women were eligible for trial screening in a lower proportion than the distribution observed in the study population (65.3% vs. 69.5% respectively). This sex-related imbalance in screening eligibility was confirmed by the distribution observed in subjects effectively screened at Fundació ACE memory clinic throughout the study period, involving a total of 53 trials (Fig. 4a). Among a total of 878 patients screened, 63% were women while the distribution in the study population was 69.5%. This uneven selection rate compared to the number of women present in the population was also was also observed in the subgroup of patients with dementia as well as in those diagnosed with MCI, but differences were less marked in the latter (Fig. 4).

## Discussion

Institutional efforts to address the gender bias in clinical research date back 25 years [32]. The US National Institutes of Health Revitalization Act of 1993 and the Sex And Gender Equity in Research guidelines issued in 2016 by the European Association of Science Editors are good examples public demands addressing gender imbalances, not only in clinical trials, but also in basic research. In 2007, gender equality policies in Spain [33] emphasized the need to address the differences between men and women in clinical trials. While these initiatives have made a difference, female underrepresentation in research remains an issue. Our study revealed that women showed a lower chance of being eligible for screening than men, and education was revealed as the main cause of this inequality.

Neuropsychological tests are commonly used instruments to determine the cognitive performance of dementia patients in clinical trials; however, they cannot be readily used in low-educated populations due to their dependence upon literacy and the need to adjust the scores by the level of education. On this basis, the exclusion of lower-educated subjects from dementia trials based on their inability for cognitive assessment is common practice. Contradictorily, AD is more prevalent among lower-educated patients, as they have been associated with a higher risk and a longer duration of the disease [17, 18]. Trial exclusion based on education becomes especially relevant among the older aged women. According to UNESCO global 2016 estimates [34], 141 million of elder adults (≥ 65 years old) remain illiterate, among which 94 million are women, still lacking basic reading and writing skills. Illiteracy is not limited to underdeveloped countries: in Spain, due to the great recession after the civil war, illiteracy rates still remain very high among people older than 65 years (109 per 1000 inhabitants [5]), with women surpassing men by 2 times (142 versus 63 per 1000 inhabitants respectively). Average elderly women are often undereducated, which limits their eligibility for clinical studies, despite the fact that AD is most prevalent in this group. Furthermore, data from AddNeuroMed study confirmed that to some extent this situation is common to other European countries. Our findings suggest that the exclusion from research based on literacy penalizes the enrolment of women, especially in dementia trials, where the average age of participants is markedly higher than in studies in prodromal Alzheimer’s disease. All in all, the large number of women with dementia from low-educated populations demands for the adaptions of these assessment tools for illiterate patients [35, 36]. The use cognitive tests validated in low-educated populations could not only improve the representativeness of women in dementia trials, but also allow for the enrolment of a more diverse sample of subjects for a higher generalizability of findings. Our findings show that education-based differences in eligibility were greater in earlier born patients, but the gap narrowed and achieved balance with increasing year of birth. This could be explained by the changing trends observed over the last few decades in educational accessibility and occupational attainment in women, which may in the foreseeable future change the specific epidemiological features of the AD population and consequentially reduce the sex bias observed in AD research.

MMSE is the most frequently applied short cognitive test for diagnosis and follow-up of MCI and AD patients. Despite its widespread use, MMSE has important limitations, including the lack of suitability for illiterate subjects, as it involves reading and writing, as well as copying and drawing on paper [37]. The use of MMSE score as a selection criterion for trial enrolment can therefore limit the eligibility of undereducated patients. This becomes especially relevant among elderly women and may further contribute imbalanced representation in research. Despite this, our results revealed MMSE as the least restrictive criterion, with only 3% of patients excluded and no significant sex-based differences. Although the MMSE score may be underestimated in low-educated patients, the lower limit score applied in our study may have only restricted the selection of functional illiterate patients, but not those with a minimal educational level. Also, we hypothesize that among individuals with a low education level, women could be more resilient than men and therefore score higher in these cognitive function tests [38]. These reasons could explain why the education-based sex gap did not translate into significant differences in terms of the MMSE score.

Despite discrepancies among studies, it is generally known that the prevalence of AD is sexually unbalanced, with women outnumbering men by 1.5 to 3 times [39]. Moreover, age is the strongest risk factor for AD and, with women’s advantage in longevity, this uneven distribution of genders increases in older aged groups leading to a markedly higher number of elderly women with AD as compared with men. Despite this, patient age greater than 85 years is a common exclusion criterion in AD trials. Accordingly, our results show that an eligible age range of 50–85 years old significantly penalized the selection of women compared with men. However, this imbalance was not present in the subgroup of patients in the prodromal stage of the disease, which may be explained by their lower average age.

According to the requirement in comorbidities, men showed a significantly lower chance of being eligible for screening than women, with a similar discriminative effect in with Alzheimer dementia and MCI. According to our data, the majority of all assessed eligibility-limiting comorbidities occur predominantly in men, which seems the reason explaining this effect. The presence of comorbidities was revealed as the most restrictive criteria for screening selection. Accordingly, a cross-sectional study of primary care elderly patients with dementia ascertained a total of 43 different comorbidities with a prevalence ranging from 44.9–1%, but showed no significant differences in the average number of comorbidities between men and women [40]. Diseases with the highest prevalence (≥ 15%) for both sexes were hypertension, anxiety and psychosis, degenerative joint disease, lipid metabolism disorders, lower back pain, and diabetes. These comorbidities are generally non-limiting for trial participation; however, the drugs used for treatment may yet be. These findings concurred with our analysis, with similar highest frequencies for osteoarthritis, hypertension, and dyslipidemia. Osteoarthritis and pain have been reported as an independent risk factor for AD and related dementia [41]. Accordingly, our results revealed remarkably elevated rates of osteoarthritis, and increased use of opioids and steroids, that were significantly more predominant in women. Osteoarthritis is not a common exclusion criterion in AD trials; however, the use of opioids and steroids are frequent causes of screening failures. Furthermore, according to previously reports [40, 42], our findings confirmed significantly higher depression rates in women, which may further contribute to sex disparities in trial eligibility. Comorbid conditions in elderly dementia patients commonly raise concerns regarding trial enrolment. On one side, comorbidities may result in study discontinuation due to barriers to appropriate care, study protocol compliance, or interactions with multiple concomitant medications. The prognosis of the study disease may interfere with comorbidities posing additional difficulties for the evaluation of response and drug tolerability. For instance, clinical trials in dementia often establish comorbid depression as an exclusion criterion, which may further contribute to the unbalanced representation of women in research. Our study highlights that the presence of comorbidities significantly discriminates men over women, which could be explained by the higher prevalence in men of the majority of disorders considered in the criterion (heart disease, alcohol abuse, chronic pulmonary disease, and kidney disorders). Depression was the single eligibility-limiting comorbidity occurring predominantly in women; however, the rate observed in men of our cohort was still markedly high (18.1%, Supplementary Table 3).

Underlying conditions also lead to polimedication, which may in turn reduce the adherence to the drug of interest and decrease its expected effects. For this reason, the use of drugs is often a limiting factor regarding clinical trial eligibility. Our findings reveal that medication limited the eligibility of up to 36% of the patients, and this requisite significantly discriminates women in the prodromal stage of AD. In order to ensure the generalizability of clinical findings more pragmatic trials with permissive trial selection criteria in terms of comorbidities and medication are needed to allow the participation of real-world elderly dementia patients.

This study analyzed factors that could explain the gender unbalances in eligibility for screening, prior to giving consent. However, beyond these criteria, men and women also differ in other socio-cultural variables that may determine their decision about trial participation or their level of engagement in the study. For instance, women typically assume the responsibilities of balancing work and family [43], which may have a negative impact on obtaining trial consent or interfere with their ability to comply with protocol visits. In fact, an estimated 71% of all dementia patients have a caregiver [20], and approximately two thirds of them are women [1]. These socio-cultural factors (such as the availability of a study partner/caregiver) were outside the scope of this study, but require further in-depth analysis and preventive measures, as they may further contribute to widen the gender gap in AD research. Another interesting point for further study is the potential effects on the gender gap of the application of digital technologies in dementia trials. The COVID-19 pandemic has led to the adaption to a model of care based on digital technologies that will continue into the recovery [44, 45]. The use of telemedicine or the application of digitized (at-home) cognitive tests are current technological developments that can benefit the participation of women in trials, as they will avoid recurrent visits to the clinic and thus facilitate family conciliation.

It is important to note that for the purpose of this study, patient characteristics, including diagnosis information, correspond to those ascertained during the initial assessment at Fundació ACE (occurring from 2008 to 2018). Therefore, we assessed patients’ screening eligibility at that single point in time. However, as these characteristics change overtime, eligibility of patients might also change. This may be especially relevant for the age, comorbidity and medication criteria. More importantly some of the patients initially diagnosed with MCI could have progressed to AD. This limitation may lead to an underestimation of the frequency of AD dementia eligible patients and an overestimation of those diagnosed with MCI. Nevertheless, the interpretation and conclusions achieved in terms of the restrictive effects of each selection criteria as well as the estimation of sex bias are expected to remain unchanged. Other limitations of our study are the single institutional and retrospective nature of its design that may limit the strength and generalizability of conclusions.

## Conclusion

In addition to its benefits in terms of screening efficiency and accuracy, the novel trial pre-screening process implemented at Fundació ACE memory clinic has proved as a valuable tool for the analysis of the population of potentially eligible candidates for trial enrolment. This selection strategy generates a list of potentially eligible candidates for trial enrolment, with heterogeneous characteristics reflecting the real-world population of patients with dementia, allowing for a detailed analysis of the factors affecting clinical trial participation.

Our study provides an estimation of the gender gap underlying in AD clinical research and points to the educational background as the main cause contributing to women underrepresentation in clinical trials. This problem becomes especially relevant among older aged women, as they often lack the basic reading and writing skills that are required for standard cognitive assessment. As equal access to education is achieved among sexes, gender unbalances in trial eligibility are likely to disappear in the near future at least in European countries; however, cultural or economic factors may lead to different scenarios in other countries. Altogether, the large number of women with dementia from low-educated populations demands for sex-focused trials with inclusive selection criteria, exempt from any influence from the patient’s literacy skills, to allow for balanced representation of women. To this end, the development of specific assessment tools suitable for the low-educated or illiterate elderly seems like a promising strategy to overcome the gender barriers in AD clinical research.

Sex-based differences were also found in the presence of comorbidities and in the use of concomitant drugs, leading to significant disparities in screening eligibility. In order to ensure the generalizability of clinical findings, pragmatic trials with more permissive or sex-focused selection criteria are needed to allow for a well-balanced representation of the real-world population of patients with AD dementia.

To conclude, this report reveals the important role that gender plays in selection for trial enrollment and brings forward the areas that demand changes in order to tackle women underrepresentation in dementia trials. However, further research is warranted in order to explore the impact of factors that were beyond the scope of this study, such as the availability of caregivers and other socio-cultural elements affecting patient’s decision about trial participation.

## Supplementary Information

**Additional file 1.**

**Additional file 2.**

**Additional file 3.**

**Additional file 4.**

**Additional file 5.**

**Additional file 6.**

## Data Availability

The datasets used and/or analyzed during the current study are available from the corresponding author on reasonable request.

## Abbreviations

AD: Alzheimer’s disease
MCI: Mild cognitive impairment
MMSE: Mini-mental state examination
NINCDS: National Institute of Neurological Disorders and Stroke
ADRDA: Alzheimer’s Disease and Related Disorders Association criteria
