# Supplementary figures and images for: The role of sex and gender in the selection of Alzheimer patients for clinical trial pre-screening
Source: Alzheimers Res Ther. 2021 May 5;13:95. doi: 10.1186/s13195-021-00833-4 (PMC8098013; doi:10.1186/s13195-021-00833-4)

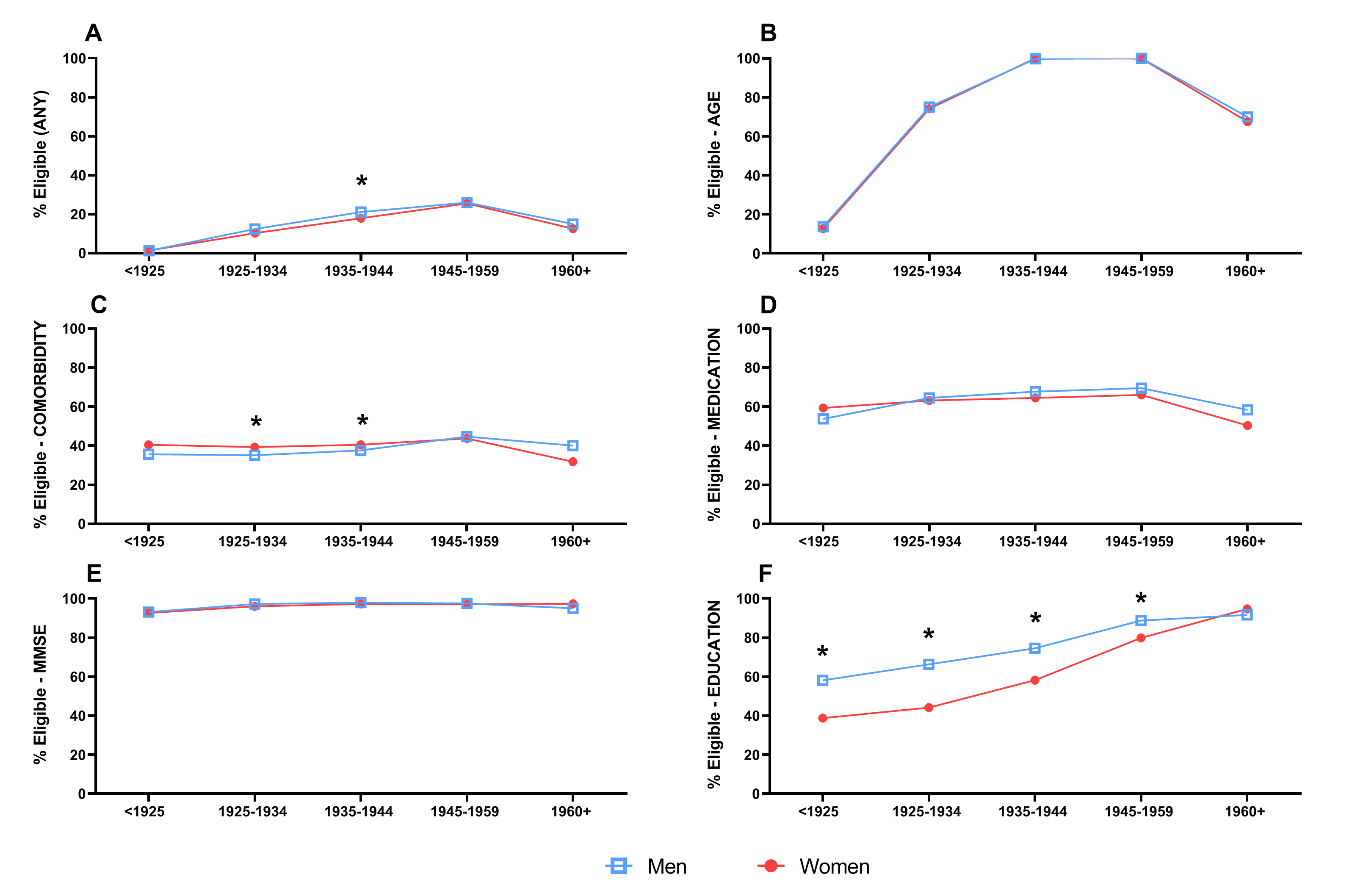

Supplement: Supplementary file 1 — Additional file 1. [file 13195_2021_833_MOESM1_ESM.tif]

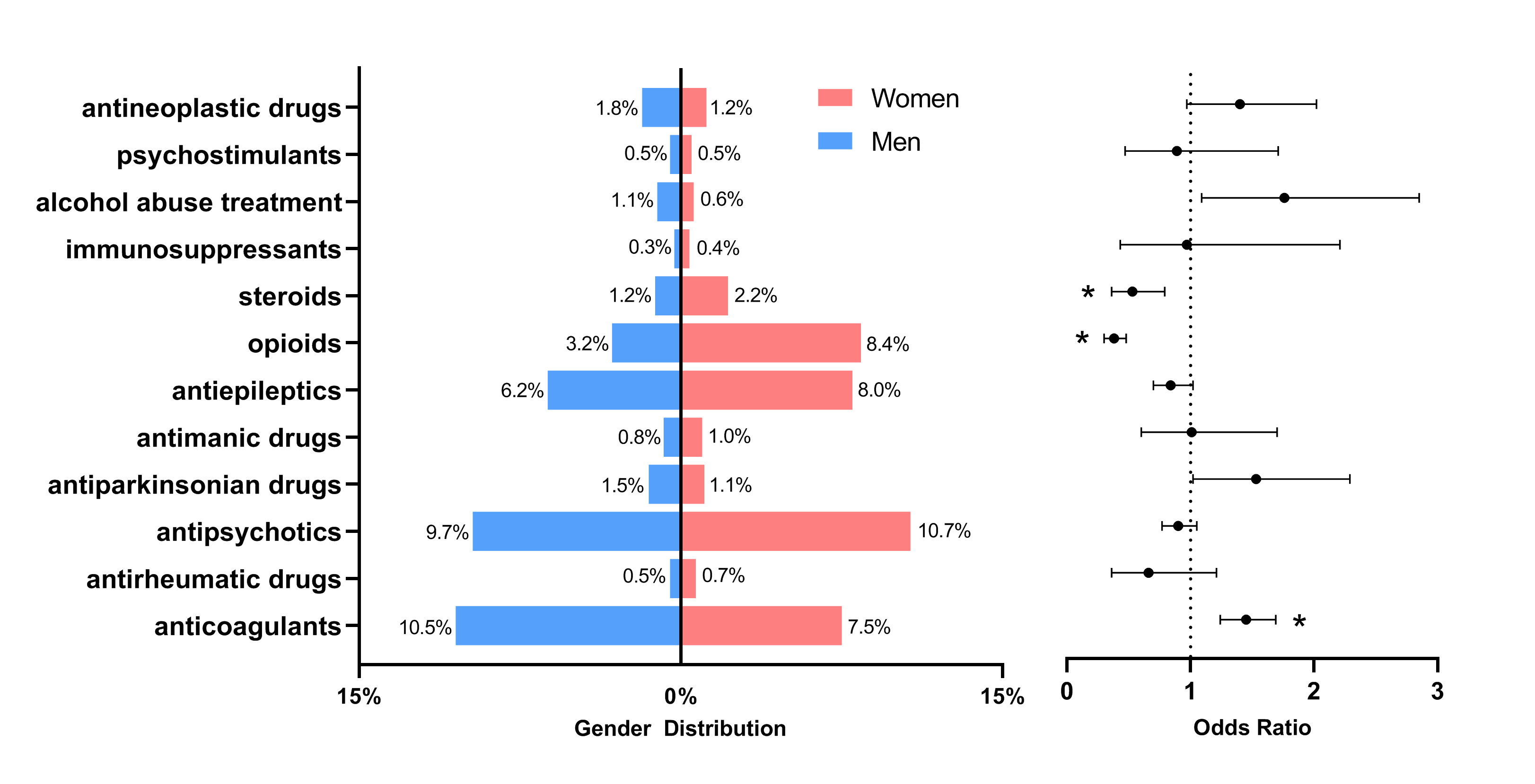

Supplement: Supplementary file 2 — Additional file 2. [file 13195_2021_833_MOESM2_ESM.tif]

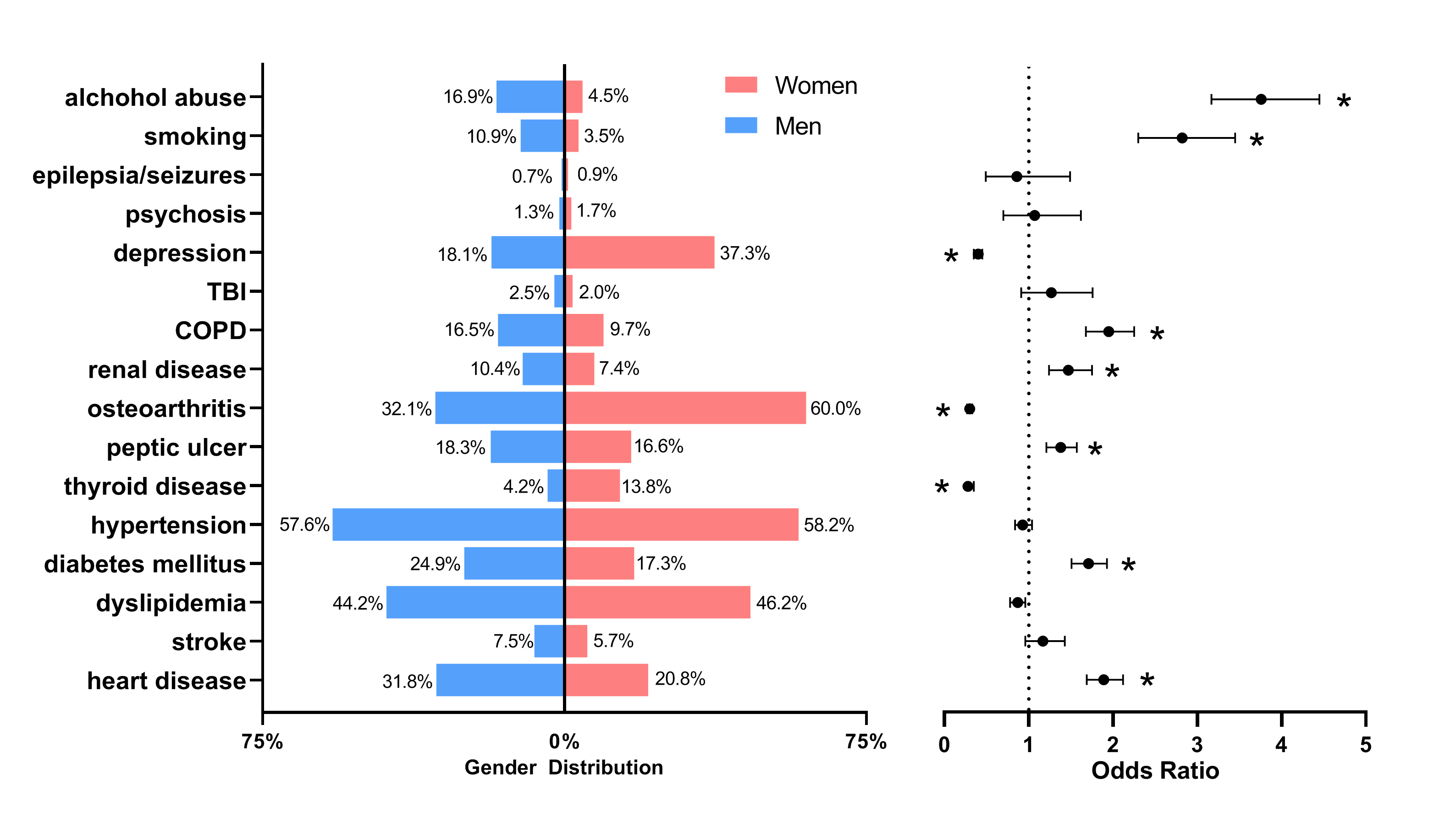

Supplement: Supplementary file 3 — Additional file 3. [file 13195_2021_833_MOESM3_ESM.tif]
