# Supplementary material for: The role of sex and gender in the selection of Alzheimer patients for clinical trial pre-screening
Source: Alzheimers Res Ther. 2021 May 5;13:95. doi: 10.1186/s13195-021-00833-4 (PMC8098013; doi:10.1186/s13195-021-00833-4)
Supplement: Supplementary file 4 — Additional file 4. [file 13195_2021_833_MOESM4_ESM.docx]

Supplementary table 1. Frequencies of AD eligible candidates for clinical trial screening by year of birth and sex.

| **AD / YOB-Gender (N)** | **Age** | **Comorbidity** | **Medication** | **MMSE** | **Education** | **All criteria** |
| --- | --- | --- | --- | --- | --- | --- |
| **<1925 (622)** | **73 (12%)** | **243 (39%)** | **345 (55%)** | **568 (91%)** | **263 (42%)*** | **8 (1%)** |
| Men (129) | 15 (12**%**) | 46 (36**%**) | 70 (54**%**) | 118 (91**%**) | 76 (59**%**) | 1 (1**%**) |
| Women (493) | 58 (12**%**) | 197 (40**%**) | 275 (56**%**) | 450 (91**%**) | 187 (38**%**) | 7 (1**%**) |
| OR [95%CI] | 1.07 [0.58-1.97] | 0.87 [0.58-1.32] | 0.96 [0.65-1.43] | 0.95 [0.47-1.92] | 2.34 [1.57-3.48] | 0.92 [0.19-4.36] |
| **1925-1934 (2,708)** | **1,863 (69%)** | **1,015 (37%)*** | **1,630 (60%)** | **2,571 (95%)** | **1,324 (49%)*** | **254 (9%)** |
| Men (757) | 533 (70**%**) | 258 (34**%**) | 448 (59**%**) | 726 (96**%**) | 486 (64**%**) | 84 (11**%**) |
| Women (1,951) | 1,330 (68**%**) | 757 (39**%**) | 1,182 (61**%**) | 1,845(95**%**) | 838 (43**%**) | 170 (9**%**) |
| OR [95%CI] | 1.19 [0.99-1.44] | 0.77 [0.64-0.93] | 0.95 [0.79-1.13] | 1.10 [0.72-1.67] | 2.43 [2.04-2.90] | 1.24 [1.00-1.53] |
| **1935-1944 (1,524)** | **1,524 (100%)** | **610 (40%)** | **951 (62%)** | **1,451 (95%)** | **884 (58%)*** | **240 (16%)*** |
| Men (428) | 428 (100**%**) | 162(38**%**) | 273 (64**%**) | 411 (96**%**) | 295 (69**%**) | 75 (18**%**) |
| Women (1,096) | 1,096 (100**%**) | 448 (41**%**) | 678 (62**%**) | 1,040 (95**%**) | 589 (54**%**) | 165 (15**%**) |
| OR [95%CI] | 1.00 [ na] | 0.85 [0.67-1.07] | 1.06 [0.84-1.34] | 1.15 [0.66-2.02] | 1.91 [1.51-2.43] | 1.23 [1.02-1.48] |
| **1945-1959 (387)** | **387 (100%)** | **217 (56%)*** | **270 (70%)** | **353 (91%)** | **292 (75%)*** | **113 (29%)** |
| Men (138) | 138 (100**%**) | 88 (64**%**) | 97 (70**%**) | 127 (92**%**) | 117 (85**%**) | 45 (33**%**) |
| Women (249) | 249 (100**%**) | 129 (52**%**) | 173 (69**%**) | 226 (91**%**) | 175 (70**%**) | 68 (27**%**) |
| OR [95%CI] | 1.00 [ na] | 1.66 [1.07-2.56] | 0.88 [0.55-1.41] | 1.34 [0.62-2.88] | 2.39 [1.38-4.14] | 1.02 [0.80-1.30] |
| **1960+ (37)** | **28 (76%)** | **21 (57%)** | **26 (70%)** | **30 (81%)** | **29 (78%)** | **8 (22%)** |
| Men (14) | 8 (57**%**) | 9 (64**%**) | 11 (79**%**) | 11 (79**%**) | 10 (71**%**) | 3 (21**%**) |
| Women (23) | 20 (87**%**) | 12 (52**%**) | 15 (65**%**) | 19 (83**%**) | 19 (83**%**) | 5 (22**%**) |
| OR [95%CI] | 0.25 [0.05-1.36] | 1.46 [0.32-6.65] | 1.64 [0.31-8.78] | 1.36 [0.06-28.45] | 0.48 [0.03-8.72] | 1.23 [0.52-2.89] |

*AD: Alzheimer’s Disease. YOB: Year of birth. MMSE: Mini-mental state examination.*

*Data are absolute frequency (relative frequency %).*

*Asterisk (*) indicate p<0.05 in test comparing eligibility between males and females by multivariable logistic regression (or univariable logistic regression for all criteria).*
