# Supplementary material for: The role of sex and gender in the selection of Alzheimer patients for clinical trial pre-screening
Source: Alzheimers Res Ther. 2021 May 5;13:95. doi: 10.1186/s13195-021-00833-4 (PMC8098013; doi:10.1186/s13195-021-00833-4)
Supplement: Supplementary file 5 — Additional file 5. [file 13195_2021_833_MOESM5_ESM.docx]

Supplementary table 2. Frequencies of MCI eligible candidates for clinical trial screening by year of birth and sex.

| **MCI / YOB-Gender (N)** | **Age** | **Comorbidity** | **Medication** | **MMSE** | **Education** | **All criteria** |
| --- | --- | --- | --- | --- | --- | --- |
| **<1925 (126)** | **24 (19%)** | **52 (41%)** | **90 (71%)*** | **126 (100%)** | **58 (46%)** | **2 (2%)** |
| Men (31) | 7 (23**%**) | 11 (35**%**) | 16 (52**%**) | 31 (100**%**) | 17 (55**%**) | 1 (3**%**) |
| Women (95) | 17 (18**%**) | 41 (43**%**) | 74 (78**%**) | 95 (100**%**) | 41 (43**%**) | 1 (1**%**) |
| OR [95%CI] | 1.27 [0.44-3.67] | 0.82 [0.34-2.00] | 0.29 [0.12-0.71] | 1.00 [ na] | 1.95 [0.82-4.65] | 0.92 [0.19-4.63] |
| **1925-1934 (1,204)** | **1,049 (87%)** | **474 (39%)** | **426 (51.6%)** | **856 (71%)** | **661 (55%)*** | **172 (14%)** |
| Men (406) | 340 (84**%**) | 150 (37**%**) | 302 (52.6**%**) | 250 (74**%**) | 285 (70**%**) | 60 (15**%**) |
| Women (798) | 709 (89**%**) | 324 (41**%**) | 294 (51.1**%**) | 554 (69**%**) | 376 (47**%**) | 112 (14**%**) |
| OR [95%CI] | 0.71 [0.50-1.01] | 0.84 [0.65-1.08] | 1.26 [0.96-1.67] | 0.74 [0.06-9.13] | 2.58 [2.00-3.33] | 1.24 [1.00-1.53] |
| **1935-1944 (1,706)** | **1,706 (100%)** | **668 (39%)** | **1,164 (68%)** | **1,697 (99%)** | **1,163 (68%)*** | **371 (22%)*** |
| Men (591) | 591 (100**%**) | 221 (37**%**) | 417 (71**%**) | 587 (99**%**) | 465 (79**%**) | 140 (24**%**) |
| Women (1,115) | 1,115 (100**%**) | 447 (40**%**) | 747 (67**%**) | 1,110 (100**%**) | 698 (63**%**) | 231 (21**%**) |
| OR [95%CI] | 1.00 [ na] | 0.83 [0.67-1.03] | 1.17 [0.93-1.46] | 0.61 [0.16-2.35] | 2.22 [1.76-2.80] | 1.23 [1.02-1.48] |
| **1945-1959 (1,105)** | **1,104 (100%)** | **440 (40%)** | **733 (66%)** | **1,098 (99%)** | **946 (86%)*** | **270 (24%)** |
| Men (386) | 386 (100**%**) | 146 (38**%**) | 267 (69**%**) | 384 (99**%**) | 348 (90**%**) | 91 (24**%**) |
| Women (719) | 718 (100**%**) | 294 (41**%**) | 466 (65**%**) | 714 (99**%**) | 598 (83**%**) | 179 (25**%**) |
| OR [95%CI] | 1.00 [ na] | 0.85 [0.65-1.09] | 1.22 [0.94-1.60] | 1.25 [0.24-6.62] | 1.87 [1.27-2.76] | 1.02 [0.80-1.30] |
| **1960+ (174)** | **116 (67%)** | **51 (29%)** | **49 (49%)** | **174 (100%)** | **169 (97%)** | **20 (11%)** |
| Men (46) | 34 (74**%**) | 15 (33**%**) | 24 (52**%**) | 46 (100**%**) | 45 (98**%**) | 6 (13**%**) |
| Women (128) | 82 (64**%**) | 36 (28**%**) | 61 (48**%**) | 128 (100**%**) | 124 (97**%**) | 14 (11**%**) |
| OR [95%CI] | 1.56 [0.73-3.33] | 1.18 [0.57-2.47] | 1.16 [0.59-2.30] | 1.00 [ na] | 1.15 [0.12-11.10] | 1.23 [0.52-2.89] |

*MCI: Mental cognitive impairment. YOB: Year of birth. MMSE: Mini-mental state examination.*

*Data are absolute frequency (relative frequency %).*

*Asterisk (*) indicate p<0.05 in test comparing eligibility between males and females by multivariable logistic regression (or univariable logistic regression for all criteria).*
