# Supplementary material for: The role of sex and gender in the selection of Alzheimer patients for clinical trial pre-screening
Source: Alzheimers Res Ther. 2021 May 5;13:95. doi: 10.1186/s13195-021-00833-4 (PMC8098013; doi:10.1186/s13195-021-00833-4)
Supplement: Supplementary file 6 — Additional file 6. [file 13195_2021_833_MOESM6_ESM.docx]

Supplementary table 3. Logistic regression p-values comparing men and women by clinical diagnosis and year of birth.

| **Diagnosis – YOB (N)** | **Age** | **Comorbidity** | **Medication** | **MMSE** | **Education** | **All criteria** |
| --- | --- | --- | --- | --- | --- | --- |
|  | p-value | p-value | p-value | p-value | p-value | p-value |
| **All (9,593)** | 0.00 | 0.00 | 0.11 | 0.52 | 0.00 | 0.00 |
| <1925 (748) | 0.53 | 0.36 | 0.22 | 0.96 | 0.00 | 0.91 |
| 1925-1934 (3,912) | 0.22 | 0.00 | 0.53 | 0.52 | 0.00 | 0.05 |
| 1935-1944 (3,230) | na | 0.03 | 0.13 | 0.61 | 0.00 | 0.03 |
| 1945-1959 (1,492) | na | 0.88 | 0.27 | 0.75 | 0.00 | 0.85 |
| 1960+ (211) | 0.72 | 0.34 | 0.37 | 0.76 | 0.68 | 0.64 |
| **AD (5,278)** | 0.01 | 0.03 | 0.77 | 0.63 | 0.00 | 0.00 |
| <1925 (622) | 0.84 | 0.52 | 0.84 | 0.88 | 0.00 | 0.91 |
| 1925-1934 (2,708) | 0.07 | 0.01 | 0.54 | 0.66 | 0.00 | 0.05 |
| 1935-1944 (1,524) | na | 0.16 | 0.62 | 0.62 | 0.00 | 0.03 |
| 1945-1959 (387) | na | 0.02 | 0.58 | 0.46 | 0.00 | 0.85 |
| 1960+ (37) | 0.11 | 0.62 | 0.56 | 0.84 | 0.62 | 0.64 |
| **MCI (4,315)** | 0.72 | 0.01 | 0.01 | 0.67 | 0.00 | 0.21 |
| <1925 (126) | 0.65 | 0.66 | 0.01 | na | 0.13 | 0.91 |
| 1925-1934 (1,204) | 0.05 | 0.17 | 0.10 | 0.81 | 0.00 | 0.05 |
| 1935-1944 (1,706) | na | 0.09 | 0.17 | 0.47 | 0.00 | 0.03 |
| 1945-1959 (1,105) | na | 0.20 | 0.14 | 0.79 | 0.00 | 0.85 |
| 1960+ (174) | 0.25 | 0.65 | 0.67 | na | 0.90 | 0.64 |

*YOB: Year of birth. MMSE: Mini-mental state examination.*

*Data are eligibility ratio (p-value).*

*Asterisk (*) indicate p<0.05 in test comparing eligibility between males and females by multivariable logistic regression (or univariable logistic regression for all criteria).*
